# Design and rationale for MICROFIT (Microvascular Coronary Rehabilitation For Improving Treatment)—a feasibility randomized trial of intensive lifestyle rehabilitation for symptomatic coronary microvascular dysfunction

**DOI:** 10.3389/fcvm.2026.1909948

**Published:** 2026-08-26

**Authors:** Joanna Abramik, David Murphy, John Graby, Anna Lacey, Theresa Smith, Christopher Lawton, Faith Martin, Fiona Gillison, Matthias G. Friedrich, Amedeo Chiribiri, Mark Mariathas, Kevin Carson, Jonathan Carl Luis Rodrigues, Dylan Thompson, Ali Khavandi

**Affiliations:** 1 Cardiac Centre, Royal United Hospitals Bath NHS Foundation Trust, Bath, United Kingdom; 2 Department for Health, University of Bath, Bath, United Kingdom; 3 Bristol Heart Institute, Bristol NHS Foundation Trust, Bristol, United Kingdom; 4 Department of Psychology, University of Bath, Bath, United Kingdom; 5 Department of Mathematical Sciences, University of Bath, Bath, United Kingdom; 6 Advanced Diagnostic Centre, St Joseph's Hospital, Newport, United Kingdom; 7 Department of Medicine, McGill University, Montreal, QC, Canada; 8 Department of Cardiovascular Imaging, King’s College London, St Thomas’ Hospital, London, United Kingdom; 9 Department of Radiology, Royal United Hospitals Bath NHS Foundation Trust, Bath, United Kingdom; 10 The HLH Imaging Group, Ruislip, United Kingdom

**Keywords:** coronary microvascular dysfunction, diet, high intensity interval training, lifestyle rehabilitation, oxygenation-sensitive cardiac MRI, quantitative stress perfusion cardiac MRI

## Abstract

**Background:**

Coronary microvascular dysfunction (CMD), a common endotype of angina with non-obstructed coronary arteries (ANOCA), is linked to poor cardiovascular outcomes, and can significantly impact quality of life, mental health, and functional capacity. CMD management strategies focus on symptom control with antianginal therapies, with limited effect and prognostic benefit. While lifestyle changes and risk factor modification are advocated by international guidelines, evidence supporting cardiac rehabilitation for CMD is sparse. It is proposed that MICROFIT—a personalized lifestyle intervention programme, utilizing exercise training and dietary counselling, underpinned by behavioural change techniques, can improve symptoms of angina in patients with CMD. This study aims to examine the feasibility of undertaking a multicenter randomized controlled trial (RCT) of the MICROFIT programme vs. usual care in patients with CMD and explore its effects on symptoms of angina.

**Design and methods:**

Forty participants (aged >18) will be recruited from two centers and randomized 1:1 to receive either MICROFIT intervention plus usual care or usual care only. The primary outcome is feasibility, measured by recruitment and retention, and adherence to the intervention. Participants' and practitioners' experience of the programme will be explored qualitatively. The secondary outcome is change in angina symptoms, to inform the design and power calculations for a future RCT. Other exploratory outcomes include changes to functional capacity (VO_2__peak_), cardiovascular MRI-derived markers of myocardial perfusion and coronary vascular function, and biomarkers of metabolic syndrome. A sub-study will explore experiences of the ANOCA diagnostic pathway from patient and healthcare professionals' perspective.

**Ethics and dissemination:**

Ethics approval was granted by the NHS Health Research Authority Plymouth and Cornwall Research Ethics Committee (reference: 24/SW/0125, 30th October 2024). Study findings will be disseminated via presentations to relevant stakeholders, national and international conferences and open-access peer-reviewed research publications.

**Clinical Trial Registration:** NCT06681896.

## 1 Introduction

Coronary microvascular dysfunction (CMD) is an increasingly recognized subtype of ischaemic heart disease (IHD), associated with elevated risks of mortality and cardiovascular morbidity (1, 2). Despite advances in diagnostic technologies, both invasive and non-invasive, and a growing understanding of the coronary microvasculature pathophysiology, CMD remains underdiagnosed and undertreated (3), with limited evidence-based management strategies (4). Current treatment approaches are largely extrapolated from those for obstructive coronary artery disease (CAD) (5, 6) and often fail to alleviate symptoms, improve quality of life, or prevent recurrent hospitalizations (7, 8).

Lifestyle interventions, including structured exercise and dietary modification, are well-established in secondary prevention of cardiovascular disease (9) and may offer specific benefits in CMD through enhancement of microvascular endothelial function (10), retardation of progression of CAD (11), or vascular remodeling (12). The microvascular endothelium plays a crucial role in maintaining vascular tone and therefore function (13). Exercise is known to enhance endothelium-dependent vasodilation of the microvasculature (10, 14), improving myocardial perfusion (15), and reducing anginal burden (16). High-intensity interval training (HIIT) is emerging as a safe, and possibly more clinically efficacious mode of cardiac rehabilitation, compared to moderate intensity exercise (17, 18). Resistance exercise has particular benefits for blood pressure control and metabolic health markers (19, 20), with the benefits particularly pronounced when combined with aerobic exercise (21). Dietary interventions likewise have proven positive effects on the endothelium (22, 23), with the Mediterranean diet being particularly beneficial (24).

However, prior studies of lifestyle interventions in CMD are limited by heterogeneity and lack of coronary function testing to enable accurate patient stratification by endotype (10, 25). MICROFIT trial's robust recruitment approach will ensure that all participants will have undergone accurate angina with non-obstructed coronary arteries (ANOCA) endotyping through gold-standard invasive coronary function testing (CFT) prior to enrolment. Changes in myocardial perfusion and coronary vascular function through quantitative stress perfusion (26–28) and oxygenation-sensitive cardiac magnetic resonance imaging (29, 30), which has recently emerged as a non-invasive method of assessment of endothelial dysfunction (31), will also be assessed at baseline and end of study.

Previously studied lifestyle interventions have often been of short duration, unidimensional (for example, exercise training only) and rarely incorporated behavioural support or targeted comorbidity management (10). To address these limitations, MICROFIT intervention has been designed as a cardiologist-led service, incorporating High Intensity Interval Training (HIIT), resistance training and diet counselling, alongside risk factor modification. The patient-centered approach is underpinned by behavioural change science and will include individually tailored exercise and dietary modifications based on personal capabilities and preferences, which are linked to improved adherence (32–35). A multidisciplinary team (MDT), consisting of a personal trainer, a dietician and a cardiologist, will provide ongoing support, gradually tapering as patient confidence and independence grow.

The hypothesis underpinning this trial is that MICROFIT intervention will improve symptoms of angina in patients with CMD diagnosed by invasive CFT. The study is aligned with the European Association for Percutaneous Coronary Intervention (EAPCI) consensus statement highlighting the urgent need for well-designed clinical trials focused on lifestyle-based strategies for CMD management (5). This trial aims to assess the feasibility of delivering a subsequent multicenter RCT, suitably powered to detect changes in angina symptom burden in patients with CMD.

## 2 Methods

### 2.1 Study design

**Figure 1:**
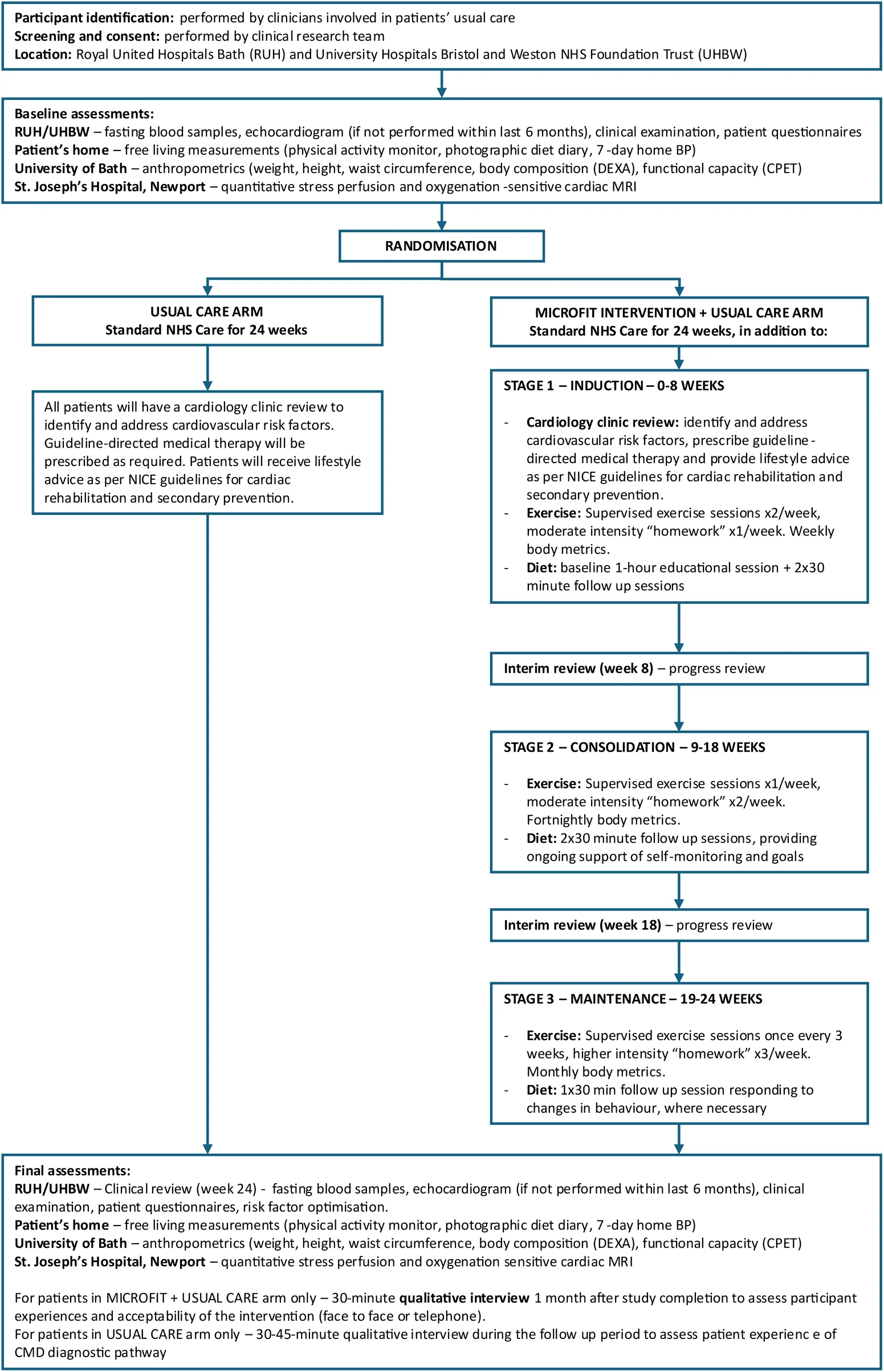
MICROFIT trial flowchart. BP, blood pressure; CPET, cardiopulmonary exercise test; CMD, coronary microvascular dysfunction; DEXA, dual-energy x-ray absorptiometry; HIIT, high intensity interval training; MRI, magnetic resonance imaging; NICE, National Institute for Health and Care Excellence.

MICROFIT is a multicenter, two parallel-arm randomized feasibility trial of Usual Care or MICROFIT intervention plus Usual Care to establish the feasibility of conducting a subsequent, adequately powered multicenter RCT. The target participant number is 40, randomized in a 1:1 fashion. The protocol follows the Standard Protocol Items: Recommendations for Interventional Trials (SPIRIT) reporting guidelines. The trial flowchart is presented in Figure 1, and schedule of assessments and procedures is presented in Supplemental Material 1.

### 2.2 Study aim

To establish the feasibility of delivering and evaluating a definitive RCT of MICROFIT with Usual Care vs. Usual Care only in the target population.

### 2.3 Study objectives and outcome measures

#### 2.3.1 Primary objectives

As a feasibility study, the primary objective is to assess the feasibility of delivering the proposed study design for a future definitive trial evaluating the efficacy of MICROFIT intervention.

Feasibility criteria assessed include:

- Recruitment rate
- Retention and adherence rates
- The acceptability of MICROFIT intervention, study design and outcome measures, assessed qualitatively from end-of-study interviews with participants and MICROFIT practitioners.

#### 2.3.2 Secondary objectives

- To assess the fidelity of the intervention offered,
- To evaluate and refine data collection procedures and outcome measures,
- To obtain preliminary data on clinical efficacy, including anginal symptom burden change measured by Seattle Angina Questionnaire-7 (SAQ-7). This will be used to guide power calculations for future trial, where it would be the primary outcome.

A qualitative sub-study involving participants in the Usual Care arm of the trial will explore the experiences of both patients and healthcare professionals of the process leading to diagnosis of CMD. It aims to provide insights on the barriers and facilitators to diagnosis and patient and healthcare professional priorities in the disease management. The details of this sub-study are presented in the Supplemental Material 2.

#### 2.3.3 Outcome measures

In line with the feasibility study primary objective, data will be collected on:

1. Recruitment rate, characterized as number of approached eligible patients consenting to trial participation
2. Retention rate, characterized as number of randomized participants who completed the trial
3. Adherence to MICROFIT intervention, assessed as attendance at supervised and unsupervised exercise sessions and dietary education sessions
4. Qualitative assessment of trial procedures and MICROFIT intervention acceptability by end-of-study interviews with practitioners and participants.

Further outcome measures collected as part of this trial include:

5. Fidelity of the intervention (structure and frequency of sessions delivered, adherence to dietary guidelines, application of behavioural change techniques)
6. Exercise dose achieved [maximum rating of perceived exertion (RPE_max_) and maximum heart rate (HR_max_) achieved during HIIT sessions, resistance training undertaken]
7. Patient self-reported angina symptom burden (SAQ-7 questionnaire)
8. Functional capacity, assessed from CPET (VO_2__peak_)
9. Myocardial perfusion and coronary vascular function, assessed from cardiac MRI
10. Patient self-reported psychological wellbeing (HADS questionnaire)
11. Patient self-reported quality of life (EQ-5D-5L questionnaire)
12. Cardiac structure and function by transthoracic echocardiography (biventricular systolic function, left ventricular size, left ventricular diastolic function, qualitative and quantitative assessment of valvular dysfunction, aortic root dimensions)
13. Serum biomarkers (fasting blood glucose and full lipid profile, HbA1c, NT-pro-BNP)
14. Body anthropometrics (height, weight, abdominal circumference)
15. Body adiposity, assessed by DEXA
16. Blood pressure control, assessed by 7-day home blood pressure diary
17. Free living activity, assessed by 7-day physical activity monitor (GeneActiv) and photographic diet diary
18. Evaluation of data collection procedures, measured as the percentage of attempted data points successfully collected.

Outcomes 6–17 will be measured at baseline and at 6 months.

### 2.4 Study population and trial settings

#### 2.4.1 Trial locations

Participants will be prospectively identified from existing clinical pathways at two recruitment sites [Royal United Hospitals Bath NHS Foundation Trust (RUH) and University Hospitals Bristol and Weston NHS Foundation Trust (UHBW)]. UHBW is a cardiac tertiary referral center and incorporates the Bristol Heart Institute. RUH is an acute, medium-sized, district general hospital (DGH). Both are specialist CMD centers and part of the NIHR-BHF cardiovascular partnership CMD Workstream. The inclusion of both a tertiary referral centre and a district general hospital was intended to evaluate the feasibility of recruitment and intervention delivery across different NHS service settings, while maintaining consistent diagnostic standards through established invasive CFT pathways. At both sites, invasive CFT is performed solely by interventional cardiologists with specialist interest and expertise in microvascular and vasomotor angina, in accordance with the consensus protocol (36).

A cardiologist from the clinical team will review participants' eligibility at both hospital sites. All participants will have undergone at least one month period of medical optimization following invasive CFT and before enrolment as standard of care. The first screening and assessment visit will take place at either hospital site. A further assessment visit, comprising cardio-pulmonary exercise testing (CPET) and dual-energy x-ray absorptiometry (DEXA), will take place at the Human Physiology Laboratory, University of Bath. The participants will also undergo cardiac MRI at St Joseph's Hospital in Newport, Wales, and will complete a 7-day home monitoring period.

The exercise component of the intervention will be delivered at local community-based exercise facilities in both Bath and Bristol, depending on patient preference. In line with PPI feedback, the dietary component of the intervention will be offered face-to-face at RUH or UHBW, virtually or over the telephone, according to patient preference. Additionally, participants will undertake unsupervised exercise in their home setting, as prescribed by their exercise trainer.

#### 2.4.2 Inclusion criteria

- Age ≥18;
- Angina or angina equivalent symptoms;
- Unobstructed coronary arteries at the time of invasive CFT. This will be defined as plaque causing <40% epicardial vessel stenosis or 40%–90% with a fractional flow reserve (FFR) ≥0.8;
- Evidence of microvascular dysfunction on CFT (CFR <2.5 and/or AChFR ≤1.5).

#### 2.4.3 Exclusion criteria

- Conditions that preclude high-intensity exercise, including:
- New York Heart Association class III/IV heart failure
- Severe left ventricular impairment (ejection fraction ≤ 35%)
- Severe heart valve disease
- Significant cardiomyopathy (as assessed by a cardiologist)
- Severe hypertension (defined as blood pressure >180/120 mmHg despite three anti-hypertensive agents)
- Uncontrolled arrhythmia
- A history of aortic dissection, recent (<6 months) acute pulmonary embolus, deep vein thrombosis, stroke or transient ischaemic attack, severe autonomic or peripheral neuropathy, acute systemic illness or fever, severe acute or chronic renal failure, severe pulmonary fibrosis or interstitial lung disease;
- Pregnancy or breastfeeding, due to excess risk related to radiation;
- Physical inability to participate in exercise;
- Significant claustrophobia or metallic implants which would limit MRI imaging;
- Intolerance to regadenoson testing (high degree atrioventricular block, severe asthma or airways disease);
- Current participation in another intervention-based trial;
- Inability to fully understand the verbal and written descriptions of the study and instructions provided during the study duration.

#### 2.4.4 Sample size

As this is a feasibility study, there is no primary outcome measure to inform a power calculation. The purpose of this feasibility study is to obtain empirical estimates to inform the design and progression criteria of the definitive multicenter trial. However, a sample size of 20 participants in each arm is sufficient to estimate feasibility outcome proportions for acceptability of 65% (90% CI: 44%–82%) and adherence rate of 85% (90% CI: 66%–96%) The corresponding intervals for recruitment rate will be more precise as the number of potential participants exceeds 40. The assumed recruitment, acceptability and adherence rates were planning assumptions informed by our local research experience from the prior *Super Rehab* trials (unpublished data), testing a similar lifestyle intervention in stable coronary artery disease and atrial fibrillation (37, 38), and published exercise intervention studies pertaining to ANOCA specifically (39–41), recognizing that this population may have a greater burden of symptoms.

A two-sided 90% confidence interval was prespecified because the principal interest in this feasibility study is the lower confidence limit for feasibility parameters such as recruitment and adherence. The lower limit of a two-sided 90% confidence interval is equivalent to a one-sided 95% confidence bound, which provides an appropriate assessment of whether these parameters are sufficiently high to support progression to a definitive trial.

### 2.5 Study proceedings

#### 2.5.1 Screening and consent

Initial pre-screening will identify potential participants who may meet inclusion criteria based on clinical information contained in the referral for invasive CFT. Potential participants will be approached on the day of their attendance for invasive CFT, once microvascular dysfunction has been confirmed as per inclusion criteria, or during a follow up clinical encounter relating to the diagnosis. Potential participants will be informed of the experimental protocol in writing and permission for contact will be sought. The potential participants will then be contacted by phone within two weeks of this initial contact and invited for a clinic assessment, at either RUH or UHBW, where they will complete screening with a cardiologist who is part of the research team. Eligible and willing participants will be asked to provide written informed consent.

#### 2.5.2 Baseline assessments

The baseline assessments will take place after consent and prior to randomization. These will include a transthoracic echocardiogram (unless already performed as part of screening), fasting blood tests (full lipid profile, glucose, HbA1c, NT-pro-BNP), body anthropometrics (height, weight, abdominal circumference), patient questionnaires [Seattle Angina Questionnaire-7 (SAQ-7), Hospital Anxiety and Depression Score (HADS) and EuroQol-5 dimensions-5 levels (EQ-5D-5L)], dual-energy x-ray absorptiometry (DEXA), and quantitative stress perfusion cardiac MRI with an additional oxygenation-sensitive sequence with hyperaemia induced by vasoactive breathing manoeuvres. Additionally, the patients will perform a 7-day blood pressure diary, alongside free-living monitoring (physical activity with GeneActiv accelerometer, and photographic diet diary) in their home setting. Demographic and other baseline characteristics data (for example, medical history or prescribed medical therapy) will be collected from patient interview and electronic medical records.

Additionally, the participants will also undertake a symptom-limited CPET to determine their VO_2peak_ and HR_max_. This test will help identify potential contraindications to high-intensity exercise and will provide guidance for exercise intensity prescription for MICROFIT HIIT sessions for patients in the intervention arm. The CPET will be conducted on a stationary cycle ergometer. Blood pressure is measured at baseline. After 5-minute warm-up against zero resistance, a subsequent ramp progression (10–20 watts/min, individualized to participant) will be applied, aiming for a total test duration of 8–12 min. Blood pressure will be monitored every 3 min. The test will be discontinued upon volitional exhaustion, targeting a Borg Rating of Perceived Exertion of ≥19, or if any absolute indications for exercise testing termination occur (42). A subsequent ≥3 min recovery phase against zero resistance will conclude the test. Participants are defined as achieving their peak VO_2_ if two or more of the following criteria are met (43):

- Heart rate ≥95% age-predicted maximum heart rate
- Respiratory exchange ratio ≥1.10;
- Rating of Perceived Exertion (RPE) ≥ 19
- Increment in VO_2_ ≤ 5 mL/kg^−1^min^−1^ in response to increasing resistance.

Participants who do not complete the CPET due to a medical contraindication noted during the exercise test will be withdrawn from the study on safety grounds. The test will be repeated if participants fail to achieve a HR_max_ > 80% of their age-predicted HR_max_ and RPE ≥19 to confirm accuracy of the result.

#### 2.5.3 Randomisation

Upon completion of baseline assessments, willing and consenting participants meeting eligibility criteria will be block randomized (1:1) to MICROFIT and Usual Care or Usual Care only. Randomization will be performed using a web-based platform (Sealed Envelope: https://www.sealedenvelope.com) in permuted blocks of four with stratification by sex, using a randomly generated allocation sequence.

### 2.6 Trial arms

#### 2.6.1 Control arm: usual care

All participants will undergo clinical assessment by a cardiologist, who will evaluate cardiovascular risk factors and initiate appropriate therapy (e.g., aspirin or statin therapy) in accordance with NICE guidelines (44). Standardised lifestyle advice will be provided verbally, including recommendations for 150 min of moderate-intensity exercise per week, a balanced diet, weight management, alcohol reduction, and smoking cessation (where applicable) in accordance with the national guidelines (44). The cardiologist will maintain overall clinical responsibility for participants during the study. General Practitioners will receive study information sheets and will be encouraged to optimize management of comorbidities (e.g., diabetes, hypertension) in line with national guidance. Upon trial completion, participants in the usual care arm will be offered a one-off session with an exercise trainer and provided with a diet information booklet consistent with that given to those in the MICROFIT intervention arm.

#### 2.6.2 Intervention arm: MICROFIT + usual care

The intervention arm participants will receive the same input from a cardiologist as participants in the usual care arm. However, the standardized lifestyle advice will be replaced with a novel intensive lifestyle intervention programme. The MICROFIT programme has been designed drawing on the research team's experience in delivery of the *Super Rehab* programme in patients with coronary artery disease (37) and atrial fibrillation (38) and is outlined in detail in Supplemental Material 3.

In summary, MICROFIT is a lifestyle intervention programme with a predominant focus on higher intensity exercise, resistance training and diet advice aligning with the latest evidence base for CMD and CAD. Behavioural change techniques incorporated into the intervention design, for example goal-setting, action-planning or self-monitoring enable and support long-term improvement in lifestyle-related risk factors. The intervention is explicitly introduced as a meaningful therapeutic option by the trial cardiologist, who provides an oversight and support to MICROFIT practitioners, and additionally undertakes interim reviews at end of each phase. These reviews will evaluate participants' progress based on data collected by the practitioners and address any remaining risk factors or participant concerns. Cardiologist-led reviews are intended to strengthen treatment credibility, validate participants' symptoms, and increase confidence in exercise and diet changes.

**Figure 2:**
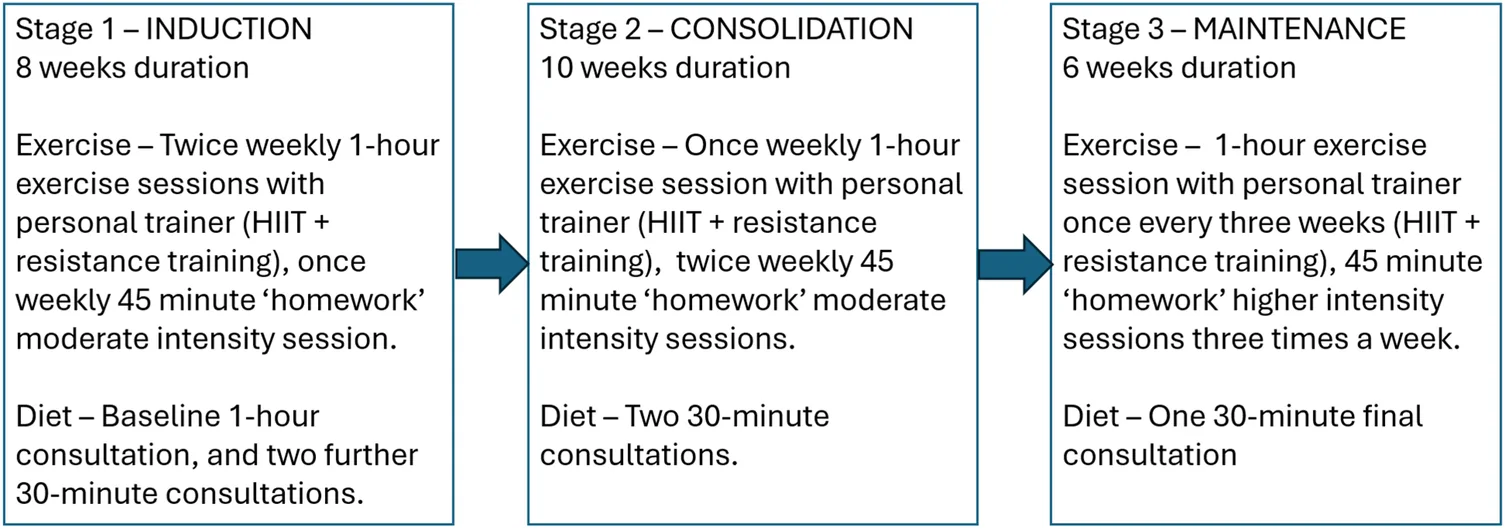
MICROFIT intervention stages. HIIT, high intensity interval training.

The MICROFIT programme consists of three phases: 1) induction (8 weeks), 2) consolidation (10 weeks) and 3) maintenance (6 weeks). These are outlined in Figure 2. The induction phase is designed to elicit rapid physiological and behavioural change, both of which are critical predictors of long-term adherence. The subsequent phases involve a progressive tapering of direct support, with the aim of fostering sustained adherence and consolidating physiological gains. The practitioners will deliver the intervention utilizing autonomy-supportive behavioural change techniques, including goal setting, action planning and self-monitoring, to encourage long term adherence.

#### 2.6.3 MICROFIT exercise training component

The exercise intervention will use the Norwegian 4 × 4 HIIT model (45), chosen for its safety, efficacy in cardiovascular disease (including CAD and heart failure) (46), and prior applications in CMD patients (25, 39). HIIT offers superior gains in peak VO_2_ (18, 47)—a strong predictor of mortality (48)—and enhances endothelial function (49) and glycocalyx thickness (50), promoting vascular health. Additionally, the repeated short bursts of high intensity exercise are suitable for deconditioned patients with cardiac and respiratory limitations, as they promote high intensity skeletal muscle training whilst limiting respiratory distress, and are achievable in the absence of substantial changes in cardiac output (51). Compared to moderate-intensity exercise, HIIT has been found to be more enjoyable (52), which may improve adherence (53). Importantly, a HIIT programme has been previously found to reassure patients with CMD that exercise is safe (54)—a concern often raised by patients, reflected in our Patient and Public Involvement (PPI) group feedback. In addition, given the benefits of resistance exercise, especially for blood pressure control and metabolic health, the sessions will conclude with twenty minutes of resistance training. The programme will follow a progressive circuit training model, rotating muscle groups between sessions to allow adequate recovery and optimize strength and functional adaptations (55). Exercise training programme will consist of supervised exercise sessions delivered in a 1:1 fashion by exercise trainers in community-based exercise facilities, and unsupervised home exercise sessions. The home exercise sessions will initially comprise of moderate-intensity exercise, progressing to high intensity in the final stage of the programme, with the addition of resistance training. The homework exercise sessions will be tailored to participants circumstances, and will be designed to use facilities and equipment already available to them.

#### 2.6.4 MICROFIT dietary component

Incorporating aspects of the Mediterranean diet alongside reduction of refined carbohydrate and sugar content and increase in healthier fats and proteins will be facilitated. Mediterranean diet has been shown to improve endothelial function with an increase in flow mediated dilatation (56, 57). Additional benefits of the intervention include a positive impact on lipid levels, insulin resistance and glycemic control (58–60) and reduction in systemic pro-inflammatory mediators (56, 61), all of which contribute to improved endothelial health.

The participants will undertake 1:1 sessions with a registered dietician, who will provide personalized advice and support upon review of pre-session combined photographic and written diet diaries. Incremental, achievable areas of improvement will be identified, with the dietician and participant collaborating to explore barriers to dietary change and potential solutions. The dietician will also continue to highlight the importance of smoking cessation, if applicable. The participants will be provided written guidance in form of an information booklet which they will be able to refer to throughout their participation in the study and beyond.

## 3 Data analysis plan

### 3.1 Blinding

The nature of the MICROFIT intervention prevents blinding of the participants and clinicians involved in their care to trial arm allocation. However, statistical and clinical outcome analyses will be completed blinded to study allocation.

### 3.2 Data collection and management

#### 3.2.1 Quantitative

Only secure NHS servers will be used to record and store patient identifiable data. Baseline and end of study data will be collected from trial assessments as per trial schedule, and it will be entered on site into an electronic case report form for each participant against participant unique identifier code, stored on NHS and University of Bath password protected computers. An electronic cross-referencing list will be stored on an NHS password protected computer database at each study site. Once collated, all data will be analyzed pseudonymously.

Assessment of the compliance with the exercise and dietary intervention will be assessed from exercise trainer and dietician records, stored on NHS password protected servers. Data will be collected regarding attendance at exercise and dietary intervention sessions, HR_max_ and RPE_max_ attained by participants during sessions, resistance training undertaken, biometric data and participants' self-reported adherence to prescribed home exercise.

##### 3.2.1.1 MRI imaging

Quantitative stress perfusion MRI imaging and Oxygenation-Sensitive MRI (OS-CMR) sequence requires image transfer off-site for analysis. The MRI imaging acquired at St Joseph's Hospital in Newport will be securely transferred via Image Exchange Portal (IEP) to the sponsor site (the RUH) for analysis. The OS-CMR image analysis will be performed in a blinded fashion in a licensed core lab, using an automated tool (OCG19, Area19 Medical Inc., Montreal). The pseudonymized dataset will be transferred securely to the core lab (CMR Core Lab at McGill University Health Centre). This data will only be used for the purposes of this study and will not be retained for any other purpose. A data sharing agreement has been established between McGill University Health Centre as the partner and RUH as the sponsor for the trial. Similar contractual arrangement is being established with the Department of Cardiovascular Imaging at King's College London for transfer and core lab analysis of pseudonymized quantitative stress perfusion sequences.

#### 3.2.2 Qualitative

Interviews with participants, practitioners delivering the MICROFIT programme, and healthcare professionals involved in CMD diagnostic pathway will be recorded, transcribed verbatim and stored on NHS servers against participant unique identifier code. All identifiable data, such as names of people or places, will be removed. Recordings will be deleted after transcription. Participants will be asked to provide consent to use verbatim quotes from the interviews in any subsequent publications.

All research records will be destroyed after ten years.

### 3.3 Data analysis

Feasibility measures, namely the proportion of participants eligible, consented, randomised, and completing follow-up, will be reported with two-sided 90% confidence intervals calculated using the exact binomial method (ignoring site and strata). In the MICROFIT + Usual Care group, the proportion of participants adequately completing the intervention (as defined by the success criteria below) will also be reported.

The distributional properties of the continuous variables will be examined using graphical methods. Participant characteristics and all outcome measures will be summarized using descriptive statistics: mean (standard deviation) for normally distributed continuous variables, median [interquartile range] for skewed continuous variables, and number (percentage) for categorical variables. For longitudinal outcomes, mean (standard deviation) absolute and percentage change from baseline will be reported separately by trial arm allocation. A two-way analysis of covariance (ANCOVA), adjusted for baseline SAQ-7 score, will be used to analyze follow-up SAQ-7 scores to provide estimates of variability and effect size to support sample size calculations for a subsequent definitive randomized controlled trial. The target treatment difference for the definitive RCT will be based on a clinically important difference in the SAQ-7 and supported by existing literature and clinical consensus. Other exploratory outcomes will be summarized descriptively to estimate variability and inform the design of a future definitive randomized controlled trial. Additional exploratory inferential analyses may be undertaken where appropriate and will be interpreted cautiously in view of the feasibility nature of the study.

### 3.4 Missing data

The analysis will be based on a complete-case per outcome basis. As this is a feasibility study, analyses of clinical outcomes are considered exploratory and will be interpreted cautiously. The extent and pattern of missing data will be described. Sensitivity analysis will be performed using multiple imputation after checking plausibility of the MAR assumption for SAQ-7 data only as the main secondary outcome which will form the basis of future power calculations.

A thematic analysis of the qualitative data collected through interviews will be undertaken.

### 3.5 Success criteria

Feasibility will be assessed according to the following success criteria:

1. Recruitment rate: ≥40% of invited eligible participants consent to participate;
2. Retention: ≥70% of the participants in both arms complete the study;
3. Intervention adherence: ≥70% of MICROFIT participants complete ≥75% of sessions;
4. Intervention acceptability: ≥70% of participants consider MICROFIT acceptable;
5. Quantitative data collection methods are feasible and acceptable to participants, with no critical issues identified within patient-reported/qualitative data that contraindicate progression to RCT;
6. Preliminary data on angina symptom-burden obtained to guide power calculations for subsequent RCT.

Criteria 1–4 will be based on the lower 90% confidence limit (CL). The criteria provide guidance regarding progression to a full trial. Progression to a full trial will be considered if all of these targets are met. If any of the progression targets are not met but fall between 60% and 100% of the target then reasons for this would be considered by the trial management and advisory groups, which may result in changes being made to the trial design for the full trial. If any of the criteria fell below 60% of the target, then progression would not occur without significant and evidence-based changes to design. The specific targets and resultant recommendations for trial progression are outlined for each criterion in Table 1. Any decision to progress to a full trial will also take into consideration evidence from other concurrent research within the field.

**Table 1:** Assessment of trial feasibility by success criteria analyzed by exact binomial method with 90% confidence interval.

Criterion	Progression to full trial	Consider progression to full trial, may need changes to design	Do not progress to full trial unless substantial change to design
	Lower limit of the 90% exact binomial confidence interval:		
Recruitment rate	≥40%	≥24%–39%	<24%
Retention rate	≥70%	≥42%–70%	<42%
Adherence rate	≥70%	≥42%–70%	<42%
Acceptability rate	≥70%	≥42%–70%	<42%

## 4 Trial management

### 4.1 Ethical approval

Ethical approval was granted by Plymouth and Cornwall Research Ethics Committee (IRAS project ID 340782, reference: 24/SW/0125, 30th October 2024). Further substantial amendment to the trial protocol (to include a qualitative sub study on patient and healthcare practitioners' experience of the diagnostic pathway to CMD) has been approved on the 28th of March 2025. Written informed consent will be required from the participants prior to enrolment in the study.

### 4.2 Study status

Recruitment for the study began on 2nd December 2024. With over three quarters of expected participants already enrolled, closure of recruitment is expected in October 2026. Follow up and participant involvement is expected to continue until May 2027.

### 4.3 Study governance

The Trial Management Group (TMG) will be led by one of the Co-Chief Investigators (AK, DT) and will comprise the full research team. The TMG will meet monthly and oversee day-to-day management of the study, including monitoring of adverse events and trial progress. A Trial Advisory Group (TAG) will take on the roles usually held by a trial steering committee and data monitoring committee and will be led by an independent consultant cardiologist, with expert input from an external consultant cardiologist with clinical and research interest in cardiac MRI imaging and exercise physiology, and PPI lead. The TAG will be joined by co-chief investigators and the study coordinator (JA) to update on progress. The TAG will be consulted for advice (e.g., regarding decisions for specific study procedures and processes, including adverse events), and will meet regularly, with a view to prepare for a subsequent multicenter RCT.

### 4.4 Adverse events

Adverse events (AEs) and serious adverse events (SAEs) will be documented throughout the study, including the final follow-up. AEs will be reviewed during TMG meetings, discussed with the TAG, and reported to the sponsor and research ethics committee in accordance with their respective guidelines.

### 4.5 Patient and public involvement (PPI)

PPI for the trial is provided by the NHS Research and Development office for Bath, Swindon and Wiltshire (BSW). Feedback from the BSW “PARTICIPATE” group and a local cardiovascular disease-focused PPI group informed development of the trial. Patient representatives contributed to refining participant-facing materials, including the participant information sheet. Ongoing PPI will be coordinated by the PPI lead, who organized engagement sessions with individuals affected by coronary artery disease or microvascular dysfunction, participates in the TAG, and supports dissemination through events and newsletters. PPI input will also inform site selection for a future RCT and the development of the definitive trial grant application.

### 4.6 Results dissemination

Upon trial completion, participants will be offered the opportunity to discuss their individual results with the study cardiologist. Anonymized trial findings will be disseminated through peer-reviewed journals, national and international conferences, and local primary and secondary care networks to raise awareness of CMD and support improved diagnosis and treatment. Results will also be shared at NHS Research & Development public dissemination events in BSW.

## 5 Discussion

MICROFIT is a feasibility randomized controlled trial of a structured lifestyle intervention comprising HIIT, resistance training and dietary counselling in patients diagnosed with CMD. The advantage of this multimodal programme over previously reported unidimensional interventions (10) is that this combination targets specific risk factors for CMD (such as obesity, sedentarism, hypertension, metabolic syndrome) and provides a comprehensive secondary prevention intervention for patients with the disease, delivered as a credible, cardiologist-led programme within a community setting. The intervention follows a progressive three-phase structure with gradually tapering levels of supervision. This design aims to facilitate the transition from supervised activity to sustainable ongoing self-management and addresses the limited duration of support that has characterised previous exercise interventions in patients with CMD (10). The programme incorporates evidence-based behaviour change techniques, which further enhances the likelihood of success and ongoing engagement beyond the programme (32).

A key strength of the MICROFIT study is its rigorous phenotyping of participants. Unlike previous lifestyle intervention studies, which have often relied on clinical suspicion of ANOCA (10, 25), eligibility requires invasive coronary function testing confirming CMD, defined by impaired coronary flow reserve (CFR <2.5) and/or acetylcholine flow reserve (AChFR ≤1.5). This diagnostic approach reduces population heterogeneity and increases confidence that any observed effects target a well-characterized population with confirmed CMD.

The multicenter design, incorporating recruitment from both a district general hospital and tertiary cardiac center, enhances the generalizability of the findings and provides an opportunity to evaluate the feasibility and deliverability of the intervention across different NHS settings, where a future definitive RCT would be set. The SAQ-7 was selected as the proposed future primary outcome because it is a validated, disease-specific measure of angina symptoms, physical limitation, and health-related quality of life (62, 63). As the principal aim of lifestyle intervention in patients with CMD is to reduce symptom burden and improve functional status, a patient-reported outcome is considered the most clinically relevant primary endpoint. Mechanistic measures, including cardiopulmonary exercise testing (64–66), quantitative stress perfusion CMR (28) and the novel OS-CMR (29, 31), will complement these findings by providing exploratory insight into the physiological mechanisms underlying any observed symptomatic benefit.

A delivery of a 24-week-long, multicenter and multidimensional intervention, alongside relatively extensive baseline and follow up investigations, does present operational challenges. The research team has substantial experience in delivery of similar lifestyle intervention programs, which helped inform the design of this study, including sample size planning (based on recruitment and attrition rates seen previously), trial locations and personnel selection and training. This extensive experience, alongside research team's engagement in national NIHR-BHF CMD Workstream network, will be of advantage for planning of the future definitive RCT.

The trial requires high levels of engagement from participants, not least due to intensity of the intervention itself, but also attendance at baseline and follow up visits. Patient and public involvement has been embedded throughout the study, informing the development of participant-facing materials, intervention delivery, and ongoing trial oversight. Informed by the PPI input, the trial strives to streamline the study visits to lessen the burden on the participants, and community and online delivery of the intervention aims to improve recruitment rates and retention. This collaborative approach is expected to improve the acceptability, relevance, and feasibility of the intervention and trial procedures.

Overall, the MICROFIT study will provide important evidence regarding the feasibility, acceptability and delivery of a comprehensive lifestyle intervention for patients with invasively confirmed CMD, while generating preliminary estimates of clinical outcomes. These findings will inform the design of a future adequately powered multicenter randomized controlled trial, aiming to address a largely unmet need of this understudied population.
