# Supplementary material for: Design and rationale for MICROFIT (Microvascular Coronary Rehabilitation For Improving Treatment)—a feasibility randomized trial of intensive lifestyle rehabilitation for symptomatic coronary microvascular dysfunction
Source: Front Cardiovasc Med. 2026 Aug 26;13:1909948. doi: 10.3389/fcvm.2026.1909948 (PMC13553230; doi:10.3389/fcvm.2026.1909948)
Supplement: Supplementary file 1 [file Supplementaryfile1.docx]

**Supplemental material 1 – Schedule of procedures and assessments**

|  | **Usual Care** | | |  | | **MICROFIT + Usual Care** | | | | | |
| --- | --- | --- | --- | --- | --- | --- | --- | --- | --- | --- | --- |
| **Procedure** | **Weeks -2 to 0** | **Week 0** | **Week 24 to 26** |  | | **Weeks -2 to 0** | **Week 0** | **Weeks 0-8** | **Weeks 9-18** | **Weeks 19-24** | **Week 24 to 26** |
| Eligibility screening | **x** |  |  |  | | **x** |  |  |  |  |  |
| Consent | **x** |  |  |  | | **x** |  |  |  |  |  |
| Baseline Assessments   - Visit 1 – echocardiography^1^, blood tests^2^, clinical examination, participant questionnaires^3^ - Visit 2 – CPET^4^, DEXA^5^, anthropometrics^6^ - Visit 3 – cardiac MRI^7^ - 7-day home monitoring^8^ | **x** |  |  |  | | **x** |  |  |  |  |  |
| Cardiology clinical review^9^ | **x** |  | **x** |  | | **x** | **x** |  |  |  |  |
| Randomisation |  | **x** |  |  | |  | **x** |  |  |  |  |
| Usual Care commences |  | **x** |  |  | |  | **x** |  |  |  |  |
| MICROFIT – Induction stage^10^ |  |  |  |  | |  |  | **x** |  |  |  |
| MICROFIT – Consolidation stage^10^ |  |  |  |  | |  |  |  | **x** |  |  |
| MICROFIT – Maintenance stage^10^ |  |  |  |  | |  |  |  |  | **x** |  |
| Practitioner Data Collection^11^ |  |  |  |  | |  |  | **x** | **x** | **x** |  |
| Cardiologist interim review^12^ |  |  |  |  | |  |  |  | **x** | **x** |  |
| End of study Assessments   - Visit 1 – echocardiography^1^, fasted blood tests^2^, clinical examination, participant questionnaires^3^ - Visit 2 – CPET^4^, DEXA^5^, anthropometrics^6^ - Visit 3 – cardiac MRI^7^ - 7-day home monitoring^8^ |  |  | **x** |  | |  |  |  |  |  | **x** |
| Interview 1 (ANOCA diagnosis experience) |  | **x^13^** | |  |  | |  |  |  |  |  |
| Interview 2 (MICROFIT experience) |  |  |  |  | |  |  |  |  |  | **x** |

^1^ LV systolic function and size, LV diastolic function, LV strain, LA strain, valvular function

^2^ Fasting glucose and lipid profile, HbA1c, NT-pro-BNP, Creatinine, eGFR

^3^ SAQ-7, EQ-5D-5L, HADS

^4^ Functional capacity assessment (determined by VO_2_peak)

^5^ Body adiposity assessment

^6^ Height, weight, BMI, abdominal circumference

^7^ cardiac structure and function, quantitative myocardial perfusion and vascular function derived from OS-CMR

^8^ 7-day home blood pressure and resting heart rate diary, diet diary and GeneActiv physical activity monitoring

^9^ Identify and address cardiovascular risk factors, prescribe guideline directed medical therapy, provide lifestyle advice as per NICE guidelines for cardiac rehabilitation and secondary prevention

^10^ As per Supplemental Material 3

^11^Practitioner Data Collection includes attendance at exercise and dietary intervention sessions, HRmax and RPEmax attained by participants during sessions, resistance training undertaken, self-monitoring data (weight, abdominal circumference, blood pressure) and participants’ self-reported adherence to prescribed home exercise.

^12^ Review of Practitioner Data Collection and discussion regarding residual concerns/cardiovascular risk factors

^13^Interview 1 can take place at any point between randomisation and end of study.
