# Supplementary material for: Design and rationale for MICROFIT (Microvascular Coronary Rehabilitation For Improving Treatment)—a feasibility randomized trial of intensive lifestyle rehabilitation for symptomatic coronary microvascular dysfunction
Source: Front Cardiovasc Med. 2026 Aug 26;13:1909948. doi: 10.3389/fcvm.2026.1909948 (PMC13553230; doi:10.3389/fcvm.2026.1909948)
Supplement: Supplementary file 2 [file Supplementaryfile2.docx]

**Patient and healthcare professionals’ perspectives on the pathway to diagnosis of coronary microvascular dysfunction – a qualitative study**

**Introduction:**

Coronary microvascular dysfunction (CMD), a variant of angina with non-obstructed coronary arteries (ANOCA), is becoming increasingly more recognised as a condition with significant implications for patients’ cardiovascular prognosis (Gdowski et al., 2020), quality of life (Gulati et al., 2023a), mental health (Rutledge et al., 2013) and economic productivity (Schumann et al., 2021). While international guidelines now recommend invasive coronary function testing for patients with ANOCA (Vrints et al., 2024), many patients report significant delays to receiving a formal diagnosis, or experience significant barriers along their diagnostic and treatment pathway (Van Schalkwijk et al., 2023).

There is limited literature describing the patient perspective of the diagnostic pathway to CMD. A recent qualitative study (Humphreys et al., 2024), highlighted experiences of dismissal, misbelief, and mistreatment among women with ischemia and no obstructive coronary artery disease (INOCA) by healthcare practitioners. However, this study was limited by its primary focus on women, with only two male participants, and its reliance on a self-selected sample from patient support groups, raising concerns about its representativeness to the broader CMD patient population. Survey studies have also illustrated patient’s experiences, with Gulati et al. (2023b) reporting that 34.4% of patients experienced symptoms for three or more years prior to receiving diagnosis, and 77.8% were told their symptoms were non-cardiac. Qualitative research has also explored the influence of receiving a definite diagnosis, informed by coronary function testing (Tseng et al., 2023), revealing a positive impact on women with INOCA, including improved healthcare experiences, quality of life, and emotional well-being. Together, these findings highlight the importance of and the necessity for more comprehensive and representative research to improve the diagnostic pathway for CMD.

There is also equally limited evidence exploring the issue from healthcare professionals’ (HCP’s) perspective. A 2019 questionnaire study of Dutch cardiologists suggests that nearly 40% of them did not recognise CMD as a separate disease entity, and 67% and 17% respectively did not think diagnostic and management strategies for CMD were evidence-based (Aribas et al., 2019). These findings were replicated in a similar study in Saudi Arabia, also highlighting differences in attitudes between cardiologists practicing in higher referral centres and general cardiology (Alanazi et al., 2019). However, no qualitative study has explored healthcare professionals’ perceptions on the pathway to CMD diagnosis or experience of managing these patients.

This qualitative study, following a hermeneutic phenomenology approach, will explore the experiences of both patients and HCPs of the process leading to diagnosis of CMD. It aims to provide valuable insights on the barriers and facilitators to diagnosis and patient and HCP priorities.

**Methods:**

Semi-structured interviews with patients with a confirmed diagnosis of CMD will be conducted, exploring patient’s diagnostic journey including a discussion of the facilitators and barriers they experienced, their interactions with healthcare practitioners and their suggestions for improving the process. The participants within the Usual Care arm of the MICROFIT trial will be approached by a member of the study team during their involvement in the study. The study will be explained to patients verbally and they will be given a patient information sheet to read. Willing and consenting participants will sign a separate consent form. Prior to the interview, participants will be offered an optional reflective workbook to consider their diagnostic experiences, with corresponding prompts to help organise these thoughts. This approach has been utilised in other qualitative studies examining the patient perspective to allow for more insightful responses during the interview (Humphreys et al., 2024). Recruitment will finish once thematic saturation is achieved and no new themes are identified.

Semi-structured interviews with healthcare professionals involved in the care of patients with ANOCA will be conducted based around a fictional scenario about a patient presenting with ANOCA (see HCP interview guide file) will be conducted. This approach has previously been applied in other studies assessing practitioner perspectives on disease management (Dixon et al., 2020, 2021), and the case study was co-developed in collaboration between an experienced cardiologist and a patient living with CMD and piloted with two senior clinicians to ensure its relevance and validity. Participants will be identified through convenience sampling (HCPs practicing in cardiology departments at sites involved in the MICROFIT trial) and further enhanced by snowballing sampling methods (to identify healthcare practitioners within the broader system, for example GPs, who have experience of managing this specific condition). Recruitment will cease once thematic saturation is reached and no new themes emerge.

The interviews will take place either remotely via telephone or videoconferencing in a private space and will be undertaken by appropriately trained members of the study team. The recordings will be taken on a digital dictaphone and audio files uploaded to NHS secure servers in a pseudonymised fashion. The recordings will be transcribed verbatim and anonymised by a member of the research team, the transcripts will be stored pseudonymously and recordings deleted after transcription. A thematic analysis of the data, utilising NVivo software, will be undertaken by the members of the study team. If direct quotes from participants are to be published, no identifiable personal information will be presented to ensure participant confidentiality is maintained.

References:

Alanazi, A., Alosaimi, M., Alkhars, A., Alghadeer, M., Alalwan, M., Altaweel, H., Alhamrani, A., Alameer, H., Alhashem, H., Alarbash, M., Alrufayi, A., Al-Shabaan, H., Almanea, K. A., Alosaimi, R. A., & Alharthi, A. (2019). *Cardiologists’ View and Management of Coronary Microvascular Disease in Clinical Practice in Saudi Arabia*.

Aribas, E., Elias-Smale, S. E., Duncker, D. J., Piek, J. J., Ikram, M. A., Appelman, Y., Roeters van Lennep, J. E., & Kavousi, M. (2019). Questionnaire survey on cardiologists’ view and management of coronary microvascular disease in clinical practice. *Netherlands Heart Journal*, *27*(5), 252–262. <https://doi.org/10.1007/s12471-019-1274-x>

Dixon, S., Hinton, L., & Ziebland, S. (2020). Supporting patients with female genital mutilation in primary care: A qualitative study exploring the perspectives of GPS’ working in England. *British Journal of General Practice*, *70*(699), E749–E756. <https://doi.org/10.3399/bjgp20X712637>

Dixon, S., McNiven, A., Talbot, A., & Hinton, L. (2021). Navigating possible endometriosis in primary care: A qualitative study of GP perspectives. In *British Journal of General Practice* (Vol. 71, Issue 710, pp. E668–E676). Royal College of General Practitioners. <https://doi.org/10.3399/BJGP.2021.0030>

Gdowski, M. A., Murthy, V. L., Doering, M., Monroy‐Gonzalez, A. G., Slart, R., & Brown, D. L. (2020). Association of Isolated Coronary Microvascular Dysfunction With Mortality and Major Adverse Cardiac Events: A Systematic Review and Meta‐Analysis of Aggregate Data. *Journal of the American Heart Association*, *9*(9). <https://doi.org/10.1161/JAHA.119.014954>

Gulati, M., Khan, N., George, M., Berry, C., Chieffo, A., Camici, P. G., Crea, F., Kaski, J. C., Marzilli, M., & Merz, C. N. B. (2023a). Ischemia with no obstructive coronary artery disease (INOCA): A patient self-report quality of life survey from INOCA international. *International Journal of Cardiology*, *371*, 28–39. <https://doi.org/10.1016/j.ijcard.2022.09.047>

Gulati, M., Khan, N., George, M., Berry, C., Chieffo, A., Camici, P. G., Crea, F., Kaski, J. C., Marzilli, M., & Merz, C. N. B. (2023b). Ischemia with no obstructive coronary artery disease (INOCA): A patient self-report quality of life survey from INOCA international. *International Journal of Cardiology*, *371*, 28–39. <https://doi.org/10.1016/J.IJCARD.2022.09.047>

Humphreys, H., Paddock, D., Brown, S., Berry, C., Cowie, A., Dawkes, S., & Nichols, S. (2024). Living with myocardial ischaemia and no obstructive coronary arteries: a qualitative study. *Open Heart*, *11*(1), e002569. <https://doi.org/10.1136/openhrt-2023-002569>

Rutledge, T., Kenkre, T. S., Bittner, V., Krantz, D. S., Thompson, D. V., Linke, S. E., Eastwood, J. A., Eteiba, W., Cornell, C. E., Vaccarino, V., Pepine, C. J., Johnson, B. D., & Bairey Merz, C. N. (2013). Anxiety associations with cardiac symptoms, angiographic disease severity, and healthcare utilization: The NHLBI-sponsored Women’s Ischemia Syndrome Evaluation. *International Journal of Cardiology*, *168*(3), 2335–2340. <https://doi.org/10.1016/j.ijcard.2013.01.036>

Schumann, C. L., Mathew, R. C., Dean, J. H. L., Yang, Y., Balfour, P. C., Shaw, P. W., Robinson, A. A., Salerno, M., Kramer, C. M., & Bourque, J. M. (2021). Functional and Economic Impact of INOCA and Influence of Coronary Microvascular Dysfunction. *JACC: Cardiovascular Imaging*, *14*(7), 1369–1379. <https://doi.org/10.1016/j.jcmg.2021.01.041>

Tseng, L. Y., Göç, N., Schwann, A. N., Cherlin, E. J., Kunnirickal, S. J., Odanovic, N., Curry, L. A., Shah, S. M., & Spatz, E. S. (2023). Illness Perception and the Impact of a Definitive Diagnosis on Women With Ischemia and No Obstructive Coronary Artery Disease: A Qualitative Study. *Circulation. Cardiovascular Quality and Outcomes*, *16*(8), 521–529. <https://doi.org/10.1161/CIRCOUTCOMES.122.009834>

Van Schalkwijk, D. L., Widdershoven, J. W. M. G., Elias-Smale, S., Hartzema-Meijer, M., Den Oudsten, B. L., Slatman, J., & Mommersteeg, P. M. C. (2023). ShareHeart: A patient journey map of patients with ischemia and non-obstructive coronary artery disease based on qualitative research. *Journal of Clinical Nursing*, *32*(13–14), 3434–3444. <https://doi.org/10.1111/jocn.16409>

Vrints, C., Andreotti, F., Koskinas, K. C., Rossello, X., Adamo, M., Ainslie, J., Banning, A. P., Budaj, A., Buechel, R. R., Chiariello, G. A., Chieffo, A., Christodorescu, R. M., Deaton, C., Doenst, T., Jones, H. W., Kunadian, V., Mehilli, J., Milojevic, M., Piek, J. J., … Shek, A. B. (2024). 2024 ESC Guidelines for the management of chronic coronary syndromes. *European Heart Journal*, *45*(36), 3415–3537. <https://doi.org/10.1093/eurheartj/ehae177>
