# Supplementary material for: Design and rationale for MICROFIT (Microvascular Coronary Rehabilitation For Improving Treatment)—a feasibility randomized trial of intensive lifestyle rehabilitation for symptomatic coronary microvascular dysfunction
Source: Front Cardiovasc Med. 2026 Aug 26;13:1909948. doi: 10.3389/fcvm.2026.1909948 (PMC13553230; doi:10.3389/fcvm.2026.1909948)
Supplement: Supplementary file 3 [file Supplementaryfile3.docx]

**MICROFIT Intervention Protocol**

**Introduction:**

MICROFIT is a lifestyle intervention designed to enable and support successful behavioural change. There is a focus on 1-on-1 delivery, higher intensity exercise, and dietary advice that aligns with the latest evidence-base for both microvascular dysfunction and broader cardiovascular disease. The MICROFIT programme has been designed drawing on the research team’s experience in delivery of the *Super Rehab* programme in patients with coronary artery disease(1) and atrial fibrillation(2).The programme will be explicitly introduced to each participant by the Cardiologist to ensure that the programme is presented as a meaningful intervention that can improve health outcomes, as there is evidence that clinician-led services may enhance participant engagement(3). From the patient perspective, the Cardiologist will be seen as leading a multidisciplinary team, including exercise trainers and dieticians, who are collectively working to improve patient’s microvascular function, measured both in terms of symptoms as well as objectively through cardiac MRI testing. Incorporating clinician delivered support for behaviour change reflects behavioural science best practice and harnesses relational continuity - the existing respected relationship that doctors already have with patients (4). The Cardiologist will introduce the concepts involved in the nutritional advice to the participant in the introductory session, provide advice and oversight to both the nutritional and exercise trainers throughout, and conduct further clinical reviews at the end of each intervention phase (Figure 1).

Figure 1 The interactions between practitioners and participants within MICROFIT intervention.

The design of the intervention relies on research evidence that sustained interventions (of up to 1 year) with frequent contact achieve more clinically meaningful results (5,6), especially where lifestyle modifications are learnt in differentiated blocks(7). In addition, exercise trainers and dieticians will use behavioural change techniques to support patient’s autonomy, including action planning, goal setting and self-monitoring, again associated with improved effectiveness of an intervention (8,9). MICROFIT thus has three phases: induction, consolidation and maintenance (Figure 2). Induction lasts 8-weeks (a well-recognised intensive-phase duration in the literature (10)), with the aim of inducing rapid physiological and behavioural change, which are key predictors of long-term success(11). Subsequent consolidation and maintenance phases constitute a tapering of direct support to engender longer-term behavioural adherence alongside consolidating physiological gains.

Figure 2 MICROFIT Intervention Schedule

**Phase 1 – *Induction (8 weeks)***

*Exercise:* Twice-weekly 1-hour exercise trainer supervised exercise sessions, with once-weekly sessions of prescribed moderate-intensity “homework”. Weekly body metrics (BP, HR, BMI) will enable goal-setting and feedback to encourage adherence.

*Diet:* Baseline 1-hour educational session with a dietician, followed by two 30-minute follow up sessions. These will be delivered virtually or face to face, dependent on participant preference.

**Phase 2 – *Consolidation (10 weeks)***

*Exercise:* Reduced supervised session frequency to once-weekly, with two sessions of prescribed moderate-intensity homework/week. Body metrics 2-weekly.

*Diet:* 2x 30-minute “*touch-base*” sessions every 5 weeks, providing ongoing behavioural support of self-monitoring and goals.

**Phase 3 - *Maintenance* *(6 weeks)***

*Exercise*: Supervised sessions reduced to one every 3 weeks, with three times/week prescribed sessions of homework (now higher-intensity aerobic exercise). Participants will be offered the option of supervised exercise sessions in this phase being undertaken virtually or continuing face-to-face. This reflects the successful expansion of virtually-delivered exercise personal training sessions seen in the COVID-19 lockdowns, and PPI feedback suggesting improved flexibility in MICROFIT delivery. Body metrics assessed at penultimate visit.

*Diet:* A 30-minute “touch-base” session responding to changes in behaviour, where necessary.

**MICROFIT Exercise Component:**

(i) *Supervised Sessions:*

Experienced exercise trainers will lead supervised 1:1 exercise sessions incorporating HIIT based on the Norwegian 4x4 model for CAD patients(12). This has been repeatedly applied in patients with CAD and heart failure (10) as well as CMD (13,14), and the evidence-base supports that HIIT is safe in appropriately screened CAD patients (10,15). A 2018 systematic review of HIIT in CAD identified no adverse cardiovascular events across 547 participants incorporating >17,000 hours of training (10). This exercise model has also been applied in two prior research studies assessing the impact of lifestyle intervention in patients with coronary artery disease (NCT05563584) and atrial fibrillation (NCT05596175) undertaken by our research group in the recent years, with no significant adverse cardiovascular events reported. At the start of each session, participants will be asked about any change in symptoms or medications and have blood pressure and heart rate measured.

Participants will be taught the Borg Rating of Perceived Exertion (RPE; 6–20) (16). The acceptable maximal heart rate will be derived from HRmax achieved at CPET.The first session will consist of a graduated, lead-in of moderate-intensity exercise prior to commencement of HIIT in subsequent sessions. Supervised sessions will use a cardiovascular exercise (e.g. static bike or treadmill) and HIIT sessions will comprise a 3-minute warm-up; four 4-minute high-intensity intervals at RPE 15 (*hard*), finishing at RPE 17-18 (*very hard*); 3 minutes of active recovery at RPE 11-13 (*somewhat hard*) between each interval; and conclude with 5 minute recovery as is recommended for cardiac patients. MyZone heart monitors will be utilised to monitor the heart rate during sessions, targeting 85-95% of HRmax in HIIT sessions, in addition to RPE rating. Trainers will help maintain target intensity and monitor participants throughout for concerning symptoms and heart rate target. To enable this and mitigate risk, all trainers will be required to complete a *MICROFIT training course* that will incorporate symptom supervision, basic life support and defibrillator training. All exercise facilities will have a defibrillator on site. HR data, final RPE, power and duration for each high-intensity interval will be recorded for subsequent workload and intensity analysis.

In addition, given the benefits of resistance exercise, especially for blood pressure control (17–19), the sessions will conclude with twenty minutes of resistance training. The programme will follow a circuit training model, rotating muscle groups between sessions to promote recovery and optimise outcomes. The *induction* phase will use gym-based equipment to develop strength and confidence. In the *consolidation* phase, the focus will shift to bodyweight and resistance band exercises, enabling participants to continue the routines independently during the *maintenance* phase. Exercises will not be rigidly prescribed, allowing trainers to tailor sessions to individual needs and capabilities. The framework and goals with regards to resistance training per phase are outlined below:

***Phase 1 - Introduction:***

- Goal: Build strength & confidence, using free-weights or weight machines & rotating between the major muscle groups.
- Begin with 10 – 15 repetitions at 40 – 50% of the 1 repetition-maximum using free weights or weight machines
- Progress to 60 – 80% of the individual’s 1 repetition-maximum after 4 weeks

***Phase 2 - Consolidation:***

- Goal: Participants to learn exercises they can do independently without gym equipment
- Mix between weights & resistance exercises against gravity / own body weight and/or use of resistance bands
- Circuits of 10 – 15 reps at 60 – 80% of the individual’s 1 repetition-maximum

***Phase 3 - Maintaining:***

- Goal: Participants to master independent resistance training exercise
- Mix between weights & resistance exercises against gravity / own body weight and/or use of resistance bands
- Circuits of 10-15 reps at 60-80% of the individual’s 1 repetition-maximum to be prescribed as home exercise alongside HIIT training sessions.

The exercise trainer will feedback data to participants as they work through the programme to highlight progress being achieved (e.g. increase in power output or virtual distance cycled). The goal will be for the participants to gradually increase their overall workload as they become more conditioned. This will be achieved by the exercise trainers increasing the bike resistance or cycling speed, whilst maintaining RPE and HR targets as prescribed.

(ii) *Homework Sessions:*

To enhance the weekly workload achieved and support long-term behaviour change, participants will be prescribed “homework” exercise sessions. This will comprise moderate-intensity aerobic exercise (RPE 13-14, HR target 60-75%) for 45-minutes in induction and consolidation phases (e.g., brisk walking), and increase to higher-intensity exercise (e.g., hill walking or jogging) in the maintenance phase. Resistance exercise using workouts learnt in the supervised session during phases 1 and 2 will be added in phase 3. Participants will be provided with written guidance on effort levels, how to monitor their symptoms, and a HR monitor to support self-directed exercise intensity, ensuring their intensity is at the appropriate level. These data will be reviewed at supervised exercise sessions by personal trainers to evaluate progress and provide support and kept for subsequent analysis.

***MICROFIT dietary component:***

Patients will complete pre-session combined photographic and written diet diaries. The nutritional advisor will work individually with the patient to identify barriers to dietary change and potential solutions. The photographic diet diary will straightforwardly help highlight incremental areas for improvement. Where indicated, the dieticians will also continue to highlight the importance of smoking cessation. The principles and guiding framework for the dietary advice will be:

- Incorporating Mediterranean-style diet into everyday lifestyle
- Focus on dietary patterns, including advice to eat at regular mealtimes and portion control rather than “calorie counting”, alongside snack reduction and identifying healthy alternatives.
- Reduced carbohydrate intake, focusing on avoidance of starch-based vegetables and refined grains
- Reduced added sugars, using diet diaries to highlight “hidden sugars”
- Drinking water (no smoothies, diet drinks, juices), unsweetened tea/coffee and alcohol in moderation
- Avoidance of ultra-processed foods, e.g., highly-processed meat or take away products
- Choosing healthy sources of protein, encouraging plant-based foods such as pulses and nuts, regular oily fish, low-fat dairy products, eggs and lean unprocessed cuts of meat (if desired)
- Avoidance of “low-fat” products, in favour of natural, whole foods with unsaturated and/or mono-saturated fats
- Increased dietary fibre, focusing on achieving this via diversifying vegetable intake, which encourages satiety, as well as cardiovascular and gastrointestinal benefits
- Reduced salt intake.

**Behavioural change support tools:**

The exercise and diet rehabilitation programme is underpinned by behavioural change science. The practitioners will receive training on the following behavioural change techniques and will be required to apply these with their participants.

- *Education*: Practitioners will reinforce the positive health outcomes that can be achieved with the suggested exercise and dietary changes the programme aims to deliver (i.e., helping patients to understand what to do, and why they are being asked to do it). This will be supported by participant’s MICROFIT booklet, outlining basic information. Prescribed ‘homework’ exercises will be individualised to each participant, taking into consideration the best way for them to incorporate these into their lifestyle.
- *Problem-solving:* Through person-centred approach and motivational interviewing, practitioners will support lifestyle changes by helping participants identify their own barriers or logistical issues to achieving their goals and how these can be overcome.
- *Social Support*: Sustaining long term change is more likely when people have support from their own networks. Participants will be asked to identify their key social support, and how they could help as part of problem solving. Participants will be encouraged to include their key social support person in dietary review sessions and homework exercise. This will help encourage and support participants, increasing the potential to maintain the lifestyle change in the longer term.
- *Goal-setting & Biofeedback:* All participants will also be set a series of longer-term goals for their involvement in the programme. However, during the programme, the practitioners will focus on shorter term, achievable, relevant goals related to the actions the participants can take and focus on between sessions that will help them achieve these longer-term goals (for example, a specific change in food type in their diet rather than the broader outcome of weight loss). MICROFIT practitioners will follow the *SMART* goal-setting principles:

Specific – set a clear target/change for participant to make (what, when & where)

Measurable – ensure progress can be assessed at future visits

Achievable – ensure the goal is something both the participant and the practitioner believe the participant is capable of achieving

Relevant – select goals that ensure a step-wise improvement

Time-bound – set a realistic time-frame for participant to achieve the goal(s) by

Changes in body metrics such as weight and abdominal waist circumference will be used

within sessions to feedback on performance – both for positive re-enforcement and to

supplement goal-setting. The 1:1 nature of the sessions will be used to develop rapport,

engender a working relationship and a level of accountability on the participant to deliver

the identified changes.

**References:**

1.	Graby J, Khavandi A, Gillison F, Smith T, Murphy D, Peacock O, et al. ‘Super Rehab’: can we achieve coronary artery disease regression? A feasibility study protocol. BMJ Open. 2023 Dec 12;13(12):e080735. doi:10.1136/bmjopen-2023-080735

2.	Murphy D, Graby J, Smith T, Peacock O, Abramik J, Antoniades C, et al. Effectiveness of a novel intervention (Super Rehab) in overweight patients with atrial fibrillation (SuRe AF): protocol for a randomised controlled trial. BMJ Open. 2025 Sep 14;15(9):e103090. doi:10.1136/bmjopen-2025-103090

3.	Chauhan BF, Jeyaraman M, Mann AS, Lys J, Skidmore B, Sibley KM, et al. Behavior change interventions and policies influencing primary healthcare professionals’ practice—an overview of reviews. Implementation Science. 2017 Dec 5;12(1):3. doi:10.1186/s13012-016-0538-8

4.	Engamba SA, Steel N, Howe A, Bachman M. Tackling multimorbidity in primary care: is relational continuity the missing ingredient? British Journal of General Practice. 2019 Feb;69(679):92–3. doi:10.3399/bjgp19X701201

5.	Dutton GR, Laitner MH, Perri MG. Lifestyle Interventions for Cardiovascular Disease Risk Reduction: A Systematic Review of the Effects of Diet Composition, Food Provision, and Treatment Modality on Weight Loss. Curr Atheroscler Rep. 2014 Oct 5;16(10):442. doi:10.1007/s11883-014-0442-0

6.	Singh N, Stewart RAH, Benatar JR. Intensity and duration of lifestyle interventions for long-term weight loss and association with mortality: a meta-analysis of randomised trials. BMJ Open. 2019 Aug 18;9(8):e029966. doi:10.1136/bmjopen-2019-029966

7.	Albert Pérez E, Poveda González M, Martínez-Espinosa RM, Molina Vila MD, Reig García-Galbis M. Practical Guidance for Interventions in Adults with Metabolic Syndrome: Diet and Exercise vs. Changes in Body Composition. Int J Environ Res Public Health. 2019 Sep 18;16(18):3481. doi:10.3390/ijerph16183481

8.	Samdal GB, Eide GE, Barth T, Williams G, Meland E. Effective behaviour change techniques for physical activity and healthy eating in overweight and obese adults; systematic review and meta-regression analyses. International Journal of Behavioral Nutrition and Physical Activity. 2017 Dec 28;14(1):42. doi:10.1186/s12966-017-0494-y

9.	Greaves CJ, Sheppard KE, Abraham C, Hardeman W, Roden M, Evans PH, et al. Systematic review of reviews of intervention components associated with increased effectiveness in dietary and physical activity interventions. BMC Public Health. 2011 Dec 18;11(1):119. doi:10.1186/1471-2458-11-119

10.	Wewege MA, Ahn D, Yu J, Liou K, Keech A. High‐Intensity Interval Training for Patients With Cardiovascular Disease—Is It Safe? A Systematic Review. J Am Heart Assoc. 2018 Nov 6;7(21). doi:10.1161/JAHA.118.009305

11.	Garber AJ, Abrahamson MJ, Barzilay JI, Blonde L, Bloomgarden ZT, Bush MA, et al. Consensus Statement by the American Association of Clinical Endocrinologists and American College of Endocrinology on the Comprehensive Type 2 Diabetes Management Algorithm – 2018 Executive Summary. Endocrine Practice. 2018 Jan;24(1):91–121. doi:10.4158/CS-2017-0153

12.	Taylor JL, Holland DJ, Spathis JG, Beetham KS, Wisløff U, Keating SE, et al. Guidelines for the delivery and monitoring of high intensity interval training in clinical populations. Prog Cardiovasc Dis. 2019 Mar;62(2):140–6. doi:10.1016/j.pcad.2019.01.004

13.	Larsen AI, Sæland C, Vegsundvåg J, Skadberg MS, Nilsen J, Butt N, et al. Aerobic high-intensity interval exercise training in patients with angina and no obstructive coronary artery disease: feasibility and physiological effects. European Heart Journal Open. 2023 Mar 2;3(2). doi:10.1093/ehjopen/oead030

14.	Oliveira J, Hoole SP, Hartley P, Farquhar M, Deaton C, Forsyth F, et al. Exercise-based cardiac rehabilitation effects on severity of angina, HRQoL and exercise capacity in patients with microvascular angina: systematic review and meta-analysis. European Journal of Cardiovascular Nursing. 2024 Jul 17;23(Supplement_1). doi:10.1093/eurjcn/zvae098.075

15.	Rognmo Ø, Moholdt T, Bakken H, Hole T, Mølstad P, Myhr NE, et al. Cardiovascular Risk of High- Versus Moderate-Intensity Aerobic Exercise in Coronary Heart Disease Patients. Circulation. 2012 Sep 18;126(12):1436–40. doi:10.1161/CIRCULATIONAHA.112.123117

16.	Borg GA. Psychophysical bases of perceived exertion. Med Sci Sports Exerc. 1982;14(5):377–81. PubMed PMID: 7154893.

17.	Braith RW, Stewart KJ. Resistance Exercise Training. Circulation. 2006 Jun 6;113(22):2642–50. doi:10.1161/CIRCULATIONAHA.105.584060

18.	Schroeder EC, Franke WD, Sharp RL, Lee D chul. Comparative effectiveness of aerobic, resistance, and combined training on cardiovascular disease risk factors: A randomized controlled trial. PLoS One. 2019 Jan 7;14(1):e0210292. doi:10.1371/journal.pone.0210292

19.	Ihalainen JK, Inglis A, Mäkinen T, Newton RU, Kainulainen H, Kyröläinen H, et al. Strength Training Improves Metabolic Health Markers in Older Individual Regardless of Training Frequency. Front Physiol. 2019 Feb 1;10. doi:10.3389/fphys.2019.00032
